# MiR-126-3p and MiR-223-3p as Biomarkers for Prediction of Thrombotic Risk in Patients with Acute Myocardial Infarction and Primary Angioplasty

**DOI:** 10.3390/jpm11060508

**Published:** 2021-06-04

**Authors:** Milan Hromadka, Zuzana Motovska, Ota Hlinomaz, Petr Kala, Frantisek Tousek, Jiri Jarkovsky, Marketa Beranova, Pavel Jansky, Michal Svoboda, Iveta Krepelkova, Richard Rokyta, Petr Widimsky, Michal Karpisek

**Affiliations:** 1 Department of Cardiology, University Hospital and Faculty of Medicine in Pilsen, Charles University, 304 60 Pilsen, Czech Republic; HROMADKA@fnplzen.cz (M.H.); ROKYTA@fnplzen.cz (R.R.); 2 Cardiocentre, Third Faculty of Medicine, Charles University and University Hospital Kralovske Vinohrady, 100 34 Prague, Czech Republic; petr.widimsky@fnkv.cz; 3 First Department of Internal Medicine—Cardioangiology, International Clinical Research Center, Faculty of Medicine, Masaryk University and St. Anne’s University Hospital, 656 91 Brno, Czech Republic; ota.hlinomaz@fnusa.cz (O.H.); marketa.beranova@fnusa.cz (M.B.); 4 Department of Internal Medicine and Cardiology, University Hospital and Faculty of Medicine, Masaryk University, 625 00 Brno, Czech Republic; pkala@fnbrno.cz; 5 Cardiocentre—Department of Cardiology, Regional Hospital, 370 01 Ceske Budejovice, Czech Republic; tousek@nemcb.cz; 6 Institute of Biostatistics and Analyses at the Faculty of Medicine and the Faculty of Science, Masaryk University, 602 00 Brno, Czech Republic; jarkovsky@iba.muni.cz (J.J.); svoboda@iba.muni.cz (M.S.); 7 Department of Anestesiology and Resuscitation, University Hospital Kralovske Vinohrady, 100 34 Prague, Czech Republic; pavel.jansky@lf3.cuni.cz; 8 BioVendor—Laboratory Medicine, 621 00 Brno, Czech Republic; krepelkova@biovendor.com (I.K.); karpisek@biovendor.com (M.K.); 9 Department of Human Pharmacology and Toxicology, Faculty of Pharmacy, University of Veterinary and Pharmaceutical Sciences, 621 00 Brno, Czech Republic

**Keywords:** acute myocardial infarction, risk stratification, microRNA, miR-126-3P, miR-223-3p, antithrombotic therapy individualization

## Abstract

**Aim.** This study was designed to evaluate the relationship between microRNAs (miRNAs), miR-126-3p and miR-223-3p, as new biomarkers of platelet activation, and predicting recurrent thrombotic events after acute myocardial infarction (AMI). **Methods and Results.** The analysis included 598 patients randomized in the PRAGUE-18 study (ticagrelor vs. prasugrel in AMI). The measurements of miRNAs were performed by using a novel miRNA immunoassay method. The association of miRNAs with the occurrence of the ischemic endpoint (EP) (cardiovascular death, nonfatal MI, or stroke) and bleeding were analyzed. The miR-223-3p level was significantly related to an increased risk of occurrence of the ischemic EP within 30 days (odds ratio (OR) = 15.74, 95% confidence interval (CI): 2.07–119.93, *p* = 0.008) and one year (OR = 3.18, 95% CI: 1.40–7.19, *p* = 0.006), respectively. The miR-126-3p to miR-223-3p ratio was related to a decreased risk of occurrence of EP within 30 days (OR = 0.14, 95% CI: 0.03–0.61, *p* = 0.009) and one year (OR = 0.37, 95% CI: 0.17–0.82, *p* = 0.014), respectively. MiRNAs were identified as independent predictors of EP even after adjustment for confounding clinical predictors. Adding miR-223-3p and miR-126-3p to miR-223-3p ratios as predictors into the model calculating the ischemic risk significantly increased the predictive accuracy for combined ischemic EP within one year more than using only clinical ischemic risk parameters. No associations between miRNAs and bleeding complications were identified. **Conclusion.** The miR-223-3p and the miR-126-3p are promising independent predictors of thrombotic events and can be used for ischemic risk stratification after AMI.

## 1. Introduction

Balancing the intensity of antiplatelet therapy according to thrombotic risk and the risk of bleeding complications is a fundamental objective when optimizing pharmacotherapy in patients after acute myocardial infarction (AMI). The limitation of established risk scores is that many predictors of ischemic events are also predictors of bleeding risk. The use of new thrombotic risk predictors has a significant clinical benefit of choosing the intensity of antithrombotic treatment, especially when they are not related to the risk of bleeding.

MicroRNAs (miRNAs) are non-coding, single-stranded molecules of RNA, 21–24 nucleotides in length. MiRNAs are not translated into proteins but nonetheless, have regulatory functions. MiRNAs can activate or inhibit various messenger RNAs at the post-transcriptional level and also affect protein synthesis at the level of translation [1]. A single specific type of miRNA can regulate the expression of an entire chain of genes, which can encode functionally or structurally related proteins [2]. The presence of miRNAs in the extracellular space allows their use in the diagnosis [3,4,5] and prognosis of patients after AMI [3,4,5]. Prospective studies in small groups of patients found an association among miRNAs and the risk of AMI, mortality, and heart failure in AMI patients [6,7]. Furthermore, several miRNAs seemed to be responsible for platelet activation and are capable of being influenced by antiplatelet therapy [8,9]. The combination of aspirin and clopidogrel or dipyridamole resulted in a decrease in miR-126, miR-150, miR-191, and miR-223 [9]. Moreover, a decreased miR-223 expression was found to be an independent predictor associated with a reduced response to clopidogrel [10].

To the best of our knowledge, no clinical study of a representative AMI patient population has been conducted to monitor the relationship of miRNAs to clearly defined clinical endpoints over the duration of dual antiplatelet therapy (DAPT).

The goal of this study was to assess the roles of miR-126-3p and miR-233-3p, as new markers of platelet activation, for predicting the recurrence of thrombotic complications in AMI patients treated with the recommended intense DAPT.

### 1.1. Methods Section

This was a genetic sub-study of the multicenter randomized PRAGUE-18 study (NCT02808767), which compared treatment with prasugrel and ticagrelor in AMI patients undergoing primary percutaneous coronary intervention (PCI). A total of 1230 patients were enrolled in the study and were monitored for one year. During the 12-month study follow-up, 40.6% of patients switched to clopidogrel. The most common reason was patient cost sharing for study drugs (73.0%), followed by the need for oral anticoagulation therapy (6.1%), adverse effects (8.3%), and other reasons (12.6%). The study did not find a difference in the primary net clinical endpoint (death, reinfarction, urgent target vessel revascularization, stroke, severe bleeding requiring transfusion, or prolonged hospitalization) within 7 days after enrollment. Analysis of the key secondary endpoint within 30 days (cardiovascular death, nonfatal myocardial infarction, or stroke) did not find a difference between prasugrel and ticagrelor in terms of efficiency and safety [11]. Furthermore, an analysis of ischemic and bleeding risk over the duration of the study indicated the safety of very early controlled de-escalation of dual antiplatelet therapy in patients with low thrombotic risk [12].

The genetic sub-study was approved by the multicenter ethics committee and the local ethics committees of the participating centers. 

### 1.2. Patients

The present sub-analysis involved a total of 598 AMI patients treated with primary PCI who were recruited from 5 of the 14 study centers. Patients signed informed consent to participate in the clinical study and genetic examination. The inclusion and exclusion criteria for the PRAGUE-18 study were described in detail in the original study [11,12]. Patients were divided into groups according to the median miR-123-3p and median miR-126-3P to miR-223-3p ratio. 

### 1.3. Laboratory Analyses

Blood collection was performed 24 ± 6 h after admission, and whole blood was kept frozen at −80 °C until analysis. EDTA plasma collection tubes were used for blood collection, and all samples were processed simultaneously in the laboratories of BioVendor—Laboratory Medicine, Brno, Czechia.

### 1.4. RNA Isolation

Determination of total miRNAs was performed in whole blood cell lysate, including platelet or leukocyte miRNAs, and free-circulating miRNAs. Key miRNA targets related to platelet function are of hematopoietic origin, and therefore, the whole blood lysate sample was selected for the laboratory analysis. The blood samples were transferred into PAXgene Blood RNA Tubes (Preanalytix, GmbH, Hombrechtikon, Switzerland) and handled according to the manufacturer’s instructions. The precipitated blood cells were solubilized with a 1.2 mL QIAzol solution (Qiagen, Valencia, CA, USA), and total RNA, including miRNAs, was isolated using a Direct-zol-96 RNA kit (Zymo Research, Irvine, CA, USA). Total RNA concentration and purity were determined using a NanoDrop ND-2000 (Thermo Fisher Scientific, Waltham, MA, USA) at wavelengths of 260 nm, 260/230 nm, and 260/280 nm.

### 1.5. MiREIA Assays

The laboratory parameters of miR-223-3p and miR-126-3p, or endogenous reference cel-miR-39-3p, miR-93-5p, miR-423-3p, and miR-150-5p, were determined using commercial MIRNA Enzyme Immunoassay (miREIA) kits (BioVendor—Laboratory Medicine, Brno, Czechia). The selection of reference miRNAs was performed using NormFinder and verified by geNorm software among four individual miRNAs or combinations thereof. 

The BioVendor’s miREIA is an enzyme immunoassay for miRNA quantification involving hybridization of miRNAs isolated from a patient’s sample to a complementary biotinylated DNA probe. MiREIA provided appropriate sensitivity, specificity, and substantially improved reproducibility over quantitative real-time polymerase chain reaction [13]. MiREIA is independent of the heparin effect.

### 1.6. MiR-126-3p to miR-223-3p Ratio

Based on the observed [14,15] opposite trends for miR-126-3p and miR-223-3p in relation to the occurrence of cardiovascular events in patients with coronary artery disease, the miR-126-3p:miR-223-3p ratio was used as a new parameter for endpoints prediction. 

### 1.7. Ischemic Risk Calculation

An increased risk of a subsequent ischemic event was defined as the presence of at least one of the following risk factors: age > 65 years, diabetes mellitus (DM) on medication, chronic kidney disease, multiple vascular involvement (peripheral artery disease, prior history of stroke), history of MI, and failed PCI [16]. 

### 1.8. Statistical Analyses

The median value was selected as a cut-off value for each miRNA based on two reasons: (i) to obtain groups with balanced and sufficient sample sizes and (ii) to apply the same consistent rule based on ranks of miRNAs. Descriptive statistics were applied in the analysis using absolute and relative frequencies for categorical variables and the median with 5th–95th percentile for continuous variables. The differences of continuous variables between two independent groups were tested by using the Mann–Whitney U test. The relationships between miRNAs; the combined ischemic endpoint (cardiovascular death, non-fatal MI, stroke); and all-cause mortality, stent thrombosis, all bleeding events, thrombolysis in myocardial infarction (TIMI) major bleeding, and Bleeding Academic Research Consortium (BARC) >3 bleeding at 30 and 365 days was computed by using logistic regression, and the effect estimates were presented by odds ratios (ORs) and their 95% confidence intervals (CIs). Multivariate logistic regression was adopted (i) for the adjustment of the miRNAs’ influences on endpoints for potential confounding characteristics of patients, including (1) ischemic endpoints: study arm, age, sex, BMI, cigarette smoking, history of hyperlipidemia, hypertension, diabetes, previous MI/PCI/coronary artery bypass grafting (CABG), chronic heart failure, chronic kidney dysfunction, periphery artery disease, BBB, symptom onset to needle time, number of diseased vessels >1, left main disease, and TIMI < 3 after PCI and (2) bleeding endpoints: age, sex, chronic kidney dysfunction, hemoglobin, creatinine, history of bleeding, and proton pump inhibitor therapy, respectively. The adjustment for covariates was performed by using the forward stepwise algorithm with only each given miRNA forced into the model. A multivariate model of ischemic factors and miRNAs was built to compare its predictive power with that of the model of ischemic risk factors only. The influence of switch to clopidogrel on evaluated endpoints was analyzed separately, such that no statistically significant influence on the results as a time-dependent covariate was found. Therefore, it was not included in further analyses using logistic regression with potential confounding factors. Receiver operating characteristic (ROC) analysis was conducted to illustrate the predictive powers of the ischemic risk factors only model and the multivariate model combining ischemic risk factors and miRNAs for the analyzed endpoints, respectively, and the area under the ROC curve (AUC) and its statistical significance were computed. These analyses were conducted by using SPSS 25 (IBM Corporation, Armonk, NY, USA).

## 2. Results

### 2.1. Patients

Patient characteristics and group descriptions based on the median miR-123-3p and median miR-126-3p to miR-223-3p ratio are summarized in Table 1.

The particular groups were homogeneous with respect to baseline characteristics, except that there was a greater proportion of males in the group with the higher miR-123-3p to miR-223-3p ratio. Primary PCI was performed in 96% of patients. The radial approach was used in 69.1% of patients. At least 1 intracoronary stent was implanted in 96.3% of patients; of the implanted stents, 73.6% were drug-eluting stents. The optimal procedural result, i.e., TIMI grade 3 flow in the infarct-related artery, was achieved in 95.3% of patients. We observed that among the patients that switched to clopidogrel, those with suboptimal coronary intervention procedures and those with bare-metal stents tended to have lower miR-223-3p levels. We did not detect any association between the time from “symptom onset to balloon” and miRNAs values.

Cardiovascular death occurred in 1.8% (*N* = 11) of patients within 30 days and in 2.7% (*N* = 16) of patients within one year. The combined ischemic endpoint occurred in 2.7% (*N* = 16) of patients within 30 days and in 5.4% (*N* = 32) of patients within one year.

### 2.2. MiRNAs and Endpoints

**Figure 1:**
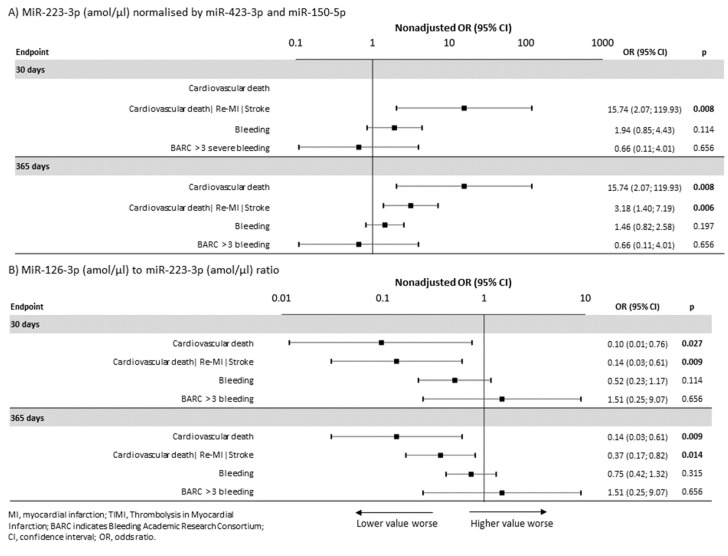
Associations of miR-223-3p (amol/µL) and the miR-126-3p to miR-223-3p ratio with the occurrences of clinical endpoints within 30 days and one year. (**A**) MiR-223-3p (amol/µL), normalized using miR-423-3p and miR-150-5p, as a predictor of endpoints. (**B**) MiR-126-3p (amol/µL) to miR-223-3p (amol/µL) ratio as a predictor of endpoints.

The normalized data for platelet-related miR-126-3p and miR-223-3p, as well as miR-1-3p connected to heart muscle tissue damage, were analyzed. The methodology is described in the Methods section. Non-normal and unique distributions were observed for all normalized data sets (Figure S1 in the Supplementary Materials). In the non-adjusted analyses, the miR-223-3p level was significantly related to the occurrence of the combined ischemic endpoint (i.e., cardiovascular death, nonfatal MI, and stroke) within 30 days (OR = 15.74, 95% CI: 2.07–119.93, *p* = 0.008) and within one year (OR = 3.18, 95% CI: 1.40–7.19, *p* = 0.006), respectively (Figure 1A). The miR-126-3p level was significantly related to the occurrence of the combined ischemic endpoint within one year (OR = 0.41, 95% CI: 0.18–0.93, *p =* 0.033) and stent thrombosis within one year (OR = 0.12, 95% CI: 0.01–0.98, *p* = 0.048), even in adjusted analyses after adjustment for confounding factors, respectively. In the non-adjusted analyses, the miR-126-3P/miR-223-3p ratio was significantly related to a decreased risk of the combined ischemic endpoint within 30 days (OR = 0.14, 95% CI: 0.03–0.61, *p* = 0.009) and within one year (OR = 0.37, 95% CI: 0.17–0.82, *p* = 0.014), respectively (Figure 1B). Therefore, both the miR-223-3p level, which is a risk factor (OR = 15.74, 95% CI2.07–119.93, *p* = 0.008), and the miR-126-3p to miR-223-3P ratio, which is a protective factor (OR = 0.14, 95% CI: 0.03 to 0.61, *p* = 0.009), were found to be significant predictors of cardiovascular events both within 30 days and within 1 year. The absolute number of patients and the percentage incidence of each endpoint relative to increased miR-223-3p values and the reduced miR-126-3P to miR-223-3p ratios are shown in Table S1A,B in the Supplementary Materials.

**Figure 2:**
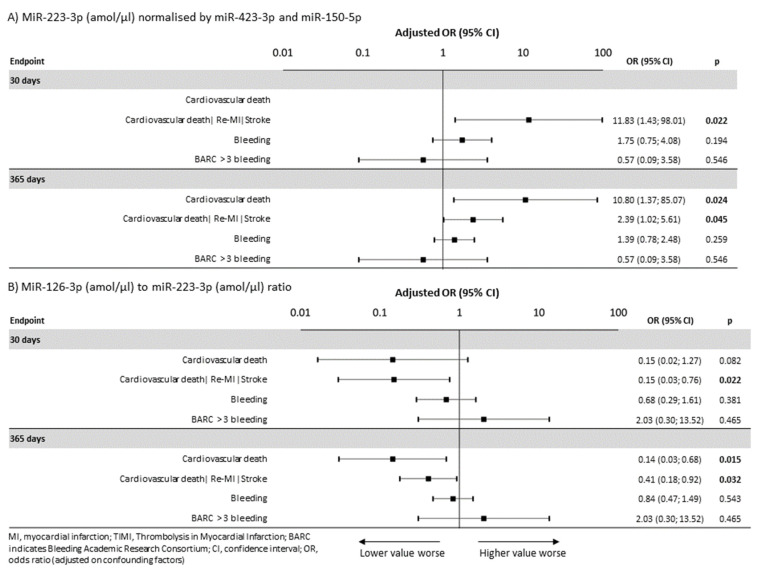
Associations of miR-223-3p (amol/µL) and the miR-126-3p to miR-223-3p ratio with the occurrences of clinical endpoints within 30 days and one year, respectively, with adjustment. (**A**) MiR-223-3p (amol/µL), normalized by miR-423-3p and miR-150-5p, as a predictor of endpoints adjusted for potential confounding factors. (**B**) The miR-126-3p (amol/µL) to miR-223-3p (amol/µL) ratio as a predictor of endpoints adjusted for potential confounding factors.

A higher incidence of the combined ischemic endpoint, even after adjustment for confounding clinical predictors (see Methods section), was significantly associated with the miR-223-3p level, both within 30 days (OR = 11.83, 95% CI: 1.47–98.01, *p* = 0.022) and within one year (OR = 2.39, 95% CI = 1.02–5.61, *p* = 0.045) (Figure 2A,B). In addition, the miR-126-3p to miR-223-3p ratio was an independently predictive factor of the risk of the combined ischemic endpoint (OR = 0.41, 95% CI: 0.18–0.93, *p* = 0.032) and definite stent thrombosis (OR = 0.09, 95% CI: 0.01–0.79, *p* = 0.030), respectively. After adjustment, the incidence of cardiovascular death was significantly associated with the miR-223-3p level (OR = 10.8, 95% CI: 1.37-85.07, *p* = 0.024) and the miR-126-3p to miR-223-3P ratio (OR = 0.14, 95% CI: 0.03–0.68, *p* = 0.015), respectively, (Figure 2A,B). 

The predictive power of the miR-126-3p to miR-223-3p ratio for the occurrence of a 30-day combined ischemic endpoint (i.e., cardiovascular death, nonfatal myocardial infarction, and stroke) within 30 days was higher than that of miR-223-3p alone, with the area under the ROC curve (AUC) being 0.741 (95% CI: 0.704–0.775) vs. 0.686 (95% CI: 0.647–0.723), respectively, and within one year, it was 0.642 (95% CI: 0.603–0.681) vs. 0.608 (95% CI: 0.567–0.647), respectively. However, differences in the AUC were not significant.

### 2.3. MiRNAs and Bleeding

The miR-223-3p values were not significantly associated with a higher risk of bleeding events within one year (OR = 1.46, 95% CI: 0.82–2.58, *p* = 0.197), with the risk of TIMI major bleeding (OR = 0.50, 95% CI: 0.05–5.23, *p* = 0.570), nor with the risk of bleeding, i.e., a BARC score above 3 (OR = 0.66, 95% CI: 0.11–4.01, *p* = 0.656). Similar patterns were observed for the miR-126-3P to miR-223-3p ratio. There was no significant relationship of the miR-126-3p to miR-223-3p ratio with the risk of any bleeding (OR = 0.75, 95% CI: 0.42–1.32, *p* = 0.315), TIMI major bleeding (OR = 2.01, 95% CI 0.18–22.25, *p* = 0.570), or a BARC score above 3 (OR = 1.51, 95% CI: 0.25–9.07, *p* = 0.656) within one year. Adjustment for potential confounding factors (see Methods section) confirmed these findings (Figure 1 and Figure 2).

### 2.4. MiRNAs and Ischemic Risk Calculation

**Figure 3:**
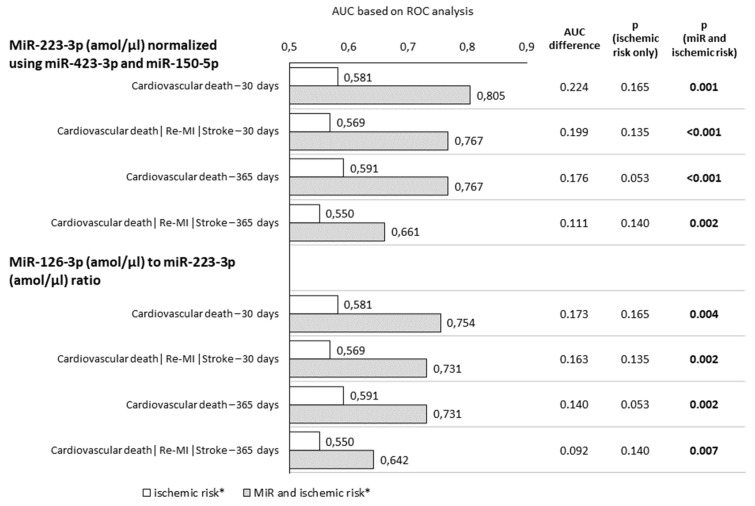
MiR-223-3p (amol/µL) and the miR-126-3p to miR-223-3p ratio and ischemic risk calculation. Adding of either miR-223-3p or miR-126-3p to miR-223-3p ratios into the model for calculating ischemic risk resulted in an increased prediction. ROC, receiver operating characteristic; AUC, area under the ROC curve. * Ischemic risk calculation according to the presence of clinical characteristics: Age > 65 years, DM, renal failure, lower limb ischemia, MI in anamnesis, multiple vessels disease, suboptimal/unsuccessful PCI.

Adding of miR-223-3p level into the model for calculating ischemic risk significantly increased the predictive accuracy for the combined ischemic endpoint within 30 days and one year more than using clinical ischemic risk parameters only (*p* < 0.001 and *p* < 0.002, respectively). Furthermore, adding of the miR-126-3p to miR-223-3p ratio led to a significant increase in the predictive accuracy for combined ischemic endpoint calculation within 30 days and one year (*p* = 0.002 and *p* = 0.007, respectively). Adding of the miR-223-3p level significantly heightened the predictive accuracy for cardiovascular death within 30 days and within one year, more than using clinical ischemic risk parameters only (*p* = 0.001). Furthermore, adding of the miR-126-3p to miR-223-3p ratio resulted in a significant raise in the predictive accuracy for cardiovascular death within 30 days and one year (*p* = 0.004 and *p* = 0.002, respectively) (Figure 3).

## 3. Discussions

The diagnostic role of miRNAs in acute coronary syndrome patients has been demonstrated in a number of recent studies [3,4,5]. It is known that some miRNAs are responsible for platelet activation and are influenced by antiplatelet therapy [8,9,10]; as such, establishing a role for miRNAs in risk stratification seems to be the next logical step in personalization of antiplatelet therapy. However, few studies have looked into this issue, and they involved small clinical cohorts; therefore, little is known about the added value of miRNAs as prospective biomarkers for risk assessment of post-MI cardiovascular events.

The correlation of elevated miR-197 and miR-223 values with cardiovascular death has been found in coronary artery disease patients [15], and increased transcoronary concentration gradients of miR-133a were significantly associated with an increased rate of death in patients after AMI within a follow-up of 32 months [7]. The serum level of miR-192 was significantly upregulated in patients with development of ischemic heart failure within 1 year after AMI [17].

MiR-233-3p in human blood is almost exclusively of platelet or megakaryocyte origin [18], and miR-126 is highly expressed in endothelial cells (ECs) and present in megakaryocytes and platelets [19]. The biological activity of miR-223-3p is related to aggregation and granule secretion [18], and miR-126-3p is associated with platelet activation [19]. However, no other cardiovascular functions have been observed, indicating the potential for high diagnostic efficacy. We examined the relationship of platelet miRNAs levels to clearly define ischemic and bleeding endpoints within a multicenter randomized study, which involved patients treated with the best evidence-based contemporary treatment strategy. Decreased miR-126-3p values and increased miR-223-3p values were associated with the incidence of cardiovascular events after AMI; therefore, the ratio of these two miRNAs can be used as a new parameter to predict endpoints.

A significant relationship between elevated miR-223-3p values and decreased miR-126-3p to miR-223-3p ratios and the occurrence of the combined ischemic endpoint, both within 30 days and one year, was demonstrated. Therefore, creation of the ratio of miR-126-3P to miR-223-3p was aimed to strengthen the effect of individual miRNAs due to their combination. After adjustment for confounding clinical predictors, selected miRNAs continued to be identified as independent predictors of ischemic events. The elevated values of miR-223-3P and reduced miR-126-3p to miR-223-3p ratios led to a significant increase in the predictive accuracy for the occurrence of combined ischemic endpoint and cardiovascular death within 1 year, rather than stratification of the major adverse cardiac events (MACE) risk according to clinical variables.

The search for the identification of new biomarkers indicative of an increased risk of ischemic complications in AMI patients is driven by the need to optimize the length and intensity of antithrombotic therapy. Effective combined antiplatelet therapy in patients after MI significantly reduces the risk of recurrent thrombotic events; however, such a therapy is associated with an increased risk of bleeding [20]. Therefore, efforts could be made to individualize the approach in post-MI patients according to each patient’s specific ischemic risk. A limitation of current clinical predictors is that they are not only associated with the risk of thrombosis, but also with the risk of bleeding [21,22,23]. Addressing the clinical imperatives of lowering the risk of bleeding while preserving ischemic benefits requires biomarkers that decouple thrombotic risk from hemorrhagic risk [24]. We did not find a significant relationship between miR-126-3p and miR-223-3p and bleeding complications; therefore, miR-126-3p and miR-223-3p can be considered as independent ischemic risk predictors. One has to mention that choosing a radial access site for PCI in STEMI patients alone reduces the risk of major bleeding and mortality compared to transfemoral access and should be the preferred approach [25]. In the PRAGUE-18 trial, choosing an access site was at the operator’s discretion, resulting in 69.1% transradial and 30.9% transfemoral procedures. This fact did not affect bleeding occurrence in the study, as there was no femoral access site-related hemorrhage.

From an analytical point of view, there are several limitations of the use of quantitative real-time polymerase chain reactions, next-generation sequencing, or microarrays techniques in routine practice. Sensitivity, specificity, and reproducibility of quantitative real-time polymerase chain reactions are generally considered very good, but an undeniable bias in the reverse transcription process has often been observed. Next-generation sequencing is a very time-consuming and expensive technique, and microarrays are usually considered for relative quantification only. A comparison of quantitative real-time polymerase chain reaction and miREIA techniques was recently published [13] and confirmed that miREIA overcomes the limitations mentioned above. MiREIA is an absolute quantitative method, which does not require reverse transcription or amplification steps, and therefore, it is a very robust, fast, as well as reproducible technology. Moreover, total assay time, including the miRNA isolation step, is less than 3 h. Additionally, absolute quantification allowed us to compare miRNA values directly or treat the data and express it as a ratio.

Heparin, an anticoagulant commonly administered in the clinical MI setting, has been a major confounding factor for the measurement of miRNA using a real-time polymerase chain reaction [26]. In our study, we presented a novel platform for miRNA determination, which was independent of heparin effects, making it superior for patients already treated with heparin.

There are, however, several barriers to introducing miRNAs as biomarkers into personalized patient care. Significant discrepancies have been observed among clinical trials, and various assays for the determination of miRNAs are still being studied within clinical diagnostics or treatment protocols. The equivocal results could be explained, at least in part, by pre-analytical and analytical variables and donor-related factors that could generate artifacts that impair accurate quantification of circulating miRNAs. Critically sensitive areas include variable sample selection, handling, and processing, as well as by blood cell contamination during sample preparation, and a lack of consensus for data normalization [13]. In this study, we used whole blood cell lysate samples; total miRNAs, including intracellular and extracellular fractions, were isolated and analyzed. It is reasonable to expect that the intra-platelet fraction was more appropriate for the assessment of antiplatelet treatment than circulating exosomes or free miRNA fractions present in serum or plasma. Our randomized study design and laboratory measurements, which were performed on a miREIA platform providing absolute quantification, also contributed to more reliable and reproducible results.

The levels of selected miRNAs corresponded to the main signals regulating platelets through miscellaneous pathways (e.g., VEGF signaling, VCAM-1, SPRED1, PIK3R2/p85-beta, P2Y_12_ receptor, and RPS6KB1/HIF1a) and exhibit diverse connections to platelet activation [8]. This could explain why miRNA values were independent parameters, associated with reduced survival due to thrombotic cardiovascular death. Furthermore, the determination of miR-126-3p and miR-223-3p corresponded to platelet signals generated in vivo, in contrast to ex vivo platelet aggregation tests. Conflicting results from ex vivo platelet aggregation tests used for guided secondary prevention antiplatelet therapy have not supported its use as a routinely recommended method [27,28].

Results of the present study advocated for using selected miRNAs for stratification of AMI patients relative to secondary prevention, according to their cardiovascular risk during hospitalization. MiRNA biomarkers associated with high platelet activation in post-AMI patients on antiplatelet therapy may define a group of patients, with acceptable risk of bleeding, that would benefit from additional use of an oral anticoagulant (e.g., low dose rivaroxaban 2.5 mg twice daily) [29,30] or prolonged dual antiplatelet therapy beyond one year with low dose ticagrelor 60mg twice daily [31,32].

In summary, our study proved that MiR-223-3p values and the miR-126-3p to miR-223-3p ratio are promising predictors of short- and long-term thrombotic events and can be used for ischemic risk stratification of patients after AMI.

## Figures and Tables

**Table 1:** Baseline characteristics of patients (*N* = 598).

	Total	miR-223-3p (amol/µl)			miR-126-3P/miR-223-3p		
Characteristics ^1^	*N* = 598	Below Median (*N* = 299)	Above Median (*N* = 299)	*p* ^2^	Below Median (*N* = 299)	Above Median (*N* = 299)	*p* ^2^
Prasugrel	294 (49.2%)	155 (51.8%)	139 (46.5%)	0.220	149 (49.8%)	145 (48.5%)	0.806
Ticagrelor	304 (50.8%)	144 (48.2%)	160 (53.5%)	0.220	150 (50.2%)	154 (51.5%)	0.806
Switch to Clopidogrel	243 (40.6%)	134 (44.8%)	109 (36.5%)	0.046	117 (39.1%)	126 (42.1%)	0.505
Age	62.0 (42.7; 78.1%)	61.9 (45; 75.9)	62.1 (42; 79.2)	0.897	62.8 (42; 78.7)	61.2 (44; 77.5)	0.105
Age > 75	54 (9.0%)	21 (7.0%)	33 (11.0%)	0.116	30 (10.0%)	24 (8.0%)	0.476
Men	465 (77.8%)	237 (79.3%)	228 (76.3%)	0.432	218 (72.9%)	247 (82.6%)	0.006
Hyperlipidemia	226 (37.8%)	122 (40.8%)	104 (34.8%)	0.152	122 (40.8%)	104 (34.8%)	0.152
BMI > 25	473 (79.1%)	236 (78.9%)	237 (79.3%)	1.000	225 (75.3%)	248 (82.9%)	0.027
Hypertension	294 (49.2%)	144 (48.2%)	150 (50.2%)	0.683	142 (47.5%)	152 (50.8%)	0.462
Current smoker	320 (53.5%)	172 (57.5%)	148 (49.5%)	0.059	153 (51.2%)	167 (55.9%)	0.286
Diabetes mellitus	124 (20.7%)	52 (17.4%)	72 (24.1%)	0.055	67 (22.4%)	57 (19.1%)	0.364
MI anamnesis	41 (6.9%)	25 (8.4%)	16 (5.4%)	0.195	21 (7.0%)	20 (6.7%)	1.000
PCI anamnesis	38 (6.4%)	18 (6.0%)	20 (6.7%)	0.867	20 (6.7%)	18 (6.0%)	0.867
CABG anamnesis	6 (1.0%)	2 (0.7%)	4 (1.3%)	0.686	4 (1.3%)	2 (0.7%)	0.686
Chronic heart failure	6 (1.0%)	5 (1.7%)	1 (0.3%)	0.216	4 (1.3%)	2 (0.7%)	0.686
Chronic renal failure	7 (1.2%)	5 (1.7%)	2 (0.7%)	0.450	3 (1.0%)	4 (1.3%)	1.000
Peripheral arterial disease	23 (3.8%)	11 (3.7%)	12 (4.0%)	1.000	13 (4.3%)	10 (3.3%)	0.672
History of bleeding	0 (0%)	0 (0%)	0 (0%)	1.000	0 (0%)	0 (0%)	1.000
STEMI	567 (94.8%)	278 (93.0%)	289 (96.7%)	0.064	283 (94.6%)	284 (95.0%)	1.000
BBB	16 (2.7%)	9 (3.0%)	7 (2.3%)	0.801	7 (2.3%)	9 (3.0%)	0.801
NSTEMI	20 (3.3%)	13 (4.3%)	7 (2.3%)	0.255	13 (4.3%)	7 (2.3%)	0.255
Bare metal stent	115 (19.2%)	46 (15.4%)	69 (23.1%)	0.022	59 (19.7%)	56 (18.7%)	0.836
Drug-eluting stent	440 (73.6%)	234 (78.3%)	206 (68.9%)	0.012	217 (72.6%)	223 (74.6%)	0.643
Bioabsorbable vascular scaffold	28 (4.7%)	14 (4.7%)	14 (4.7%)	1.000	16 (5.4%)	12 (4.0%)	0.562
TIMI after PCI < 3	28 (4.7%)	7 (2.3%)	21 (7.0%)	0.011	16 (5.4%)	12 (4.0%)	0.562
Number of diseased vessels >1	281 (47.0%)	138 (46.2%)	143 (47.8%)	0.743	152 (50.8%)	129 (43.1%)	0.071
Left main disease	16 (2.7%)	9 (3.0%)	7 (2.3%)	0.801	9 (3.0%)	7 (2.3%)	0.801
Suboptimal or unsuccessful PCI	25 (4.2%)	4 (1.3%)	21 (7.0%)	<0.001	16 (5.4%)	9 (3.0%)	0.220
Time to hospital	2.5 (0.8; 24.0%)	2.8 (1; 23.0)	2.4 (1; 24.0)	0.098	2.7 (1; 28.0)	2.5 (1; 16.4)	0.561
Time to hospital >3 h	222 (41.3%)	118 (44.4%)	104 (38.4%)	0.162	120 (44.3%)	102 (38.3%)	0.189
Time to hospital >6 h	109 (20.3%)	56 (21.1%)	53 (19.6%)	0.670	63 (23.2%)	46 (17.3%)	0.107
Discharge, n (%)							
Aspirin	578 (96.7%)	294 (98.3%)	284 (95.0%)	0.038	286 (95.7%)	292 (97.7%)	0.255
β-Blockers	494 (82.6%)	248 (82.9%)	246 (82.3%)	0.914	246 (82.3%)	248 (82.9%)	0.914
ACE inhibitors	463 (77.4%)	243 (81.3%)	220 (73.6%)	0.031	221 (73.9%)	242 (80.9%)	0.050
ARBs	42 (7.0%)	23 (7.7%)	19 (6.4%)	0.632	21 (7.0%)	21 (7.0%)	1.000
Statins	563 (94.1%)	284 (95.0%)	279 (93.3%)	0.486	283 (94.6%)	280 (93.6%)	0.728
Proton pump inhibitors	374 (62.5%)	197 (65.9%)	177 (59.2%)	0.108	181 (60.5%)	193 (64.5%)	0.353

^1^ absolute and relative frequencies for categorical variables and median supplemented by 5th–95th percentile for continuous variables. ^2^ Fisher exact test for categorical variables and Mann–Whitney U test for continuous variables. ACE, angiotensin-converting-enzyme; ARB, angiotensin receptor blocker; BMI, body mass index; MI, myocardial infarction; PCI, percutaneous coronary intervention; CABG, coronary artery bypass grafting; STEMI, ST-segment–elevation myocardial infarction; BBB, bundle-branch block; NSTEMI, non–ST-segment–elevation myocardial infarction; TIMI, thrombolysis in myocardial infarction.

## Data Availability

All data are available upon request.

## Supplementary Materials

The following are available online at https://www.mdpi.com/article/10.3390/jpm11060508/s1, Figure S1: Distribution of miRNA concentration values: charts show the shape of statistical distribution of measured miRNA concentration with individual scale of axis for each chart, Table S1A: MiR-223-3p (amol/µl) normalised by (miR-423-3p a miR-150-5p); Table S1B: MiR-126-3p (amol/µl) to miR-223-3p (amol/µl) ratio.
