# Supplementary material for: MiR-126-3p and MiR-223-3p as Biomarkers for Prediction of Thrombotic Risk in Patients with Acute Myocardial Infarction and Primary Angioplasty
Source: J Pers Med. 2021 Jun 4;11(6):508. doi: 10.3390/jpm11060508 (PMC8230013; doi:10.3390/jpm11060508)
Supplement: Supplementary file 1 [file jpm-11-00508-s001.zip › jpm-1202782-supplementary.pdf]

MiR-126-3p and MiR-223-3p as Biomarkers for Prediction of Thrombotic Risk in Patients with Acute Myocardial Infarction and Primary Angioplasty.

The PRAGUE-18 Genetic Substudy.

Hromadka, et al.

SUPPLEMENTARY DATA

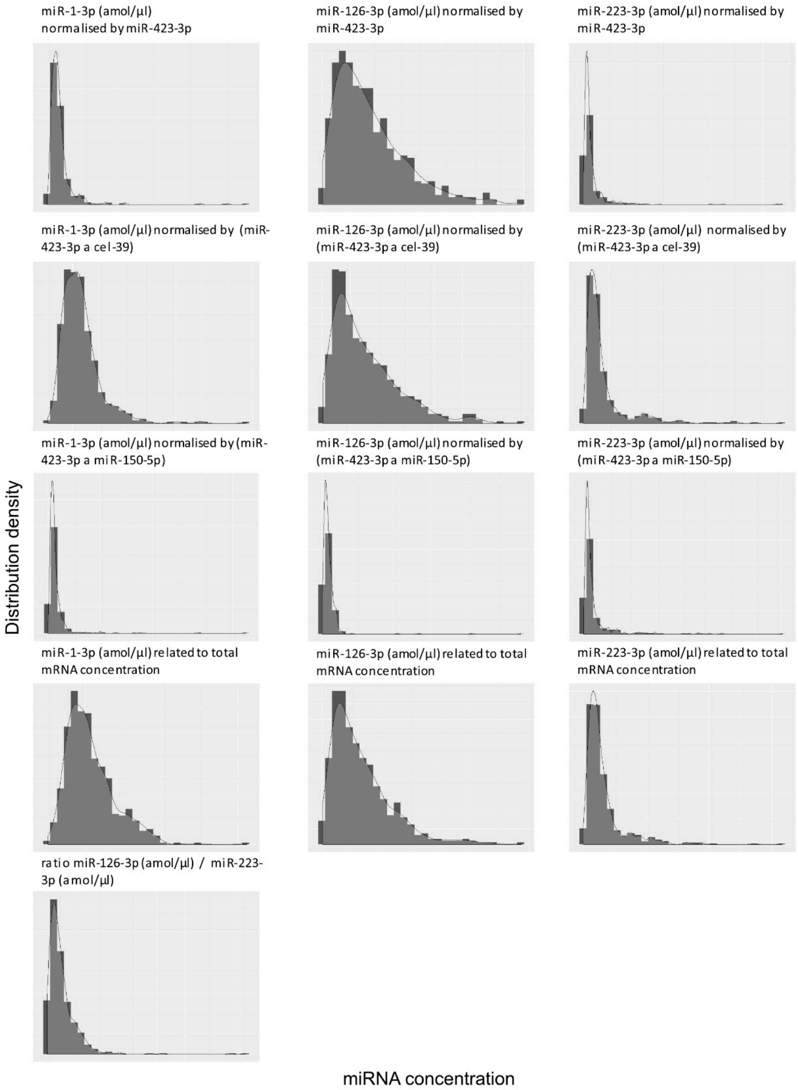
miR-1-3p (amol/µl) normalised by miR-423-3p
miR-126-3p (amol/µl) normalised by miR-423-3p
miR-223-3p (amol/µl) normalised by miR-423-3p
miR-1-3p (amol/µl) normalised by (miR-423-3p a cel-39)
miR-126-3p (amol/µl) normalised by (miR-423-3p a cel-39)
miR-223-3p (amol/µl) normalised by (miR-423-3p a cel-39)
miR-1-3p (amol/µl) normalised by (miR-423-3p a miR-150-5p)
miR-126-3p (amol/µl) normalised by (miR-423-3p a miR-150-5p)
miR-223-3p (amol/µl) normalised by (miR-423-3p a miR-150-5p)
miR-1-3p (amol/µl) related to total mRNA concentration
miR-126-3p (amol/µl) related to total mRNA concentration
miR-223-3p (amol/µl) related to total mRNA concentration
ratio miR-126-3p (amol/µl) / miR-223-3p (amol/µl)
Distribution density
miRNA concentration

Supplementary Figure 1 Distribution of miRNA concentration values: charts show the shape of statistical distribution of measured miRNA concentration with individual scale of axis for each chart

The data in our study demonstrate the importance of the normalization strategy when investigating miRNAs in blood. Four miRNAs were selected from publications for data normalization: cel-miR-39-3p, miR-93-5p, miR-150-5p and miR-423-3p. Among them, geNorm suggested using the combination of miR-423 and cell-39 (M-value 0.75), while NormFinder ranked individual miRNA by stability values as follows: miR-150 (0.198) < 423 (0.222) < 39 (0.452) < 93 (0.592). A stability value of 0.2 or less is generally accepted for use in data normalization. Overall, the combinations of miR-150 and 423 (NormFinder), miR-423-3p and cel-miR-39-3p (geNorm), individual miR-423-3p (both NormFinder and geNorm), and additionally the total RNA concentration and the miR-126-3p/miR-223-3p ratio were selected for data normalization.

Table S_1A. MiR-223-3p (amol/µl) normalised by (miR-423-3p a miR-150-5p)

	Below median[1]	Above median[1]	Nonadjusted OR (95% CI)[2]	p[2]
**30 days**				
Cardiovascular death	0 (0.0%)	11 (3.7%)		
Re-MI	0 (0.0%)	6 (2.0%)		
Stroke	1 (0.3%)	0 (0.0%)		
Cardiovascular death| Re-MI |Stroke	1 (0.3%)	15 (5.0%)	15.739 (2.066; 119.932)	**0.008**
Mortality	0 (0.0%)	11 (3.7%)		
Stent thrombosis	0 (0.0%)	6 (2.0%)		
Bleeding	9 (3.0%)	17 (5.7%)	1.942 (0.852; 4.430)	0.114
TIMI - major bleeding	2 (0.7%)	1 (0.3%)	0.498 (0.045; 5.525)	0.570
BARC > 3 bleeding	3 (1.0%)	2 (0.7%)	0.664 (0.110; 4.005)	0.656
**365 days**				
Cardiovascular death	1 (0.3%)	15 (5.0%)	15.739 (2.066; 119.932)	**0.008**
Re-MI	6 (2.0%)	11 (3.7%)	1.865 (0.681; 5.110)	0.225
Stroke	2 (0.7%)	1 (0.3%)	0.498 (0.045; 5.525)	0.570
Cardiovascular death| Re-MI |Stroke	8 (2.7%)	24 (8.0%)	3.175 (1.402; 7.186)	**0.006**
Mortality	3 (1.0%)	19 (6.4%)	6.695 (1.960; 22.873)	**0.002**

Stent thrombosis	2 (0.7%)	8 (2.7%)	4.082 (0.860; 19.387)	0.077
Bleeding	22 (7.4%)	31 (10.4%)	1.456 (0.822; 2.579)	0.197
TIMI - major bleeding	2 (0.7%)	1 (0.3%)	0.498 (0.045; 5.525)	0.570
BARC > 3 bleeding	3 (1.0%)	2 (0.7%)	0.664 (0.110; 4.005)	0.656

[1] absolute and relative frequencies for categorical variables

[2] odds ratio and its statistical significance

MI, myocardial infarction; PCI, percutaneous coronary intervention; TIMI, Thrombolysis in Myocardial Infarction; BARC indicates Bleeding Academic Research Consortium.

Table S_1B. MiR-126-3p (amol/µl) to miR-223-3p (amol/µl) ratio

	Below median[1]	Above median[1]	Nonadjusted OR (95% CI)[2]	p[2]
**30 days**				
Cardiovascular death	10 (3.3%)	1 (0.3%)	0.097 (0.012; 0.762)	**0.027**
Re-MI	5 (1.7%)	1 (0.3%)	0.197 (0.023; 1.699)	0.140
Stroke	1 (0.3%)	0 (0.0%)		
Cardiovascular death| Re-MI |Stroke	14 (4.7%)	2 (0.7%)	0.137 (0.031; 0.609)	**0.009**
Mortality	10 (3.3%)	1 (0.3%)	0.097 (0.012; 0.762)	**0.027**
Stent thrombosis	5 (1.7%)	1 (0.3%)	0.197 (0.023; 1.699)	0.140
Bleeding	17 (5.7%)	9 (3.0%)	0.515 (0.226; 1.174)	0.114
TIMI - major bleeding	1 (0.3%)	2 (0.7%)	2.007 (0.181; 22.250)	0.570
BARC > 3 bleeding	2 (0.7%)	3 (1.0%)	1.505 (0.250; 9.073)	0.656
**365 days**				
Cardiovascular death	14 (4.7%)	2 (0.7%)	0.137 (0.031; 0.609)	**0.009**
Re-MI	10 (3.3%)	7 (2.3%)	0.693 (0.260; 1.845)	0.463
Stroke	2 (0.7%)	1 (0.3%)	0.498 (0.045; 5.525)	0.570

Cardiovascular death| Re-MI |Stroke	23 (7.7%)	9 (3.0%)	0.372 (0.169; 0.819)	**0.014**
Mortality	16 (5.4%)	6 (2.0%)	0.362 (0.140; 0.939)	**0.037**
Stent thrombosis	7 (2.3%)	3 (1.0%)	0.423 (0.108; 1.651)	0.215
Bleeding	30 (10.0%)	23 (7.7%)	0.747 (0.423; 1.319)	0.315
TIMI - major bleeding	1 (0.3%)	2 (0.7%)	2.007 (0.181; 22.250)	0.570
BARC > 3 bleeding	2 (0.7%)	3 (1.0%)	1.505 (0.250; 9.073)	0.656

[1] absolute and relative frequencies for categorical variables

[2] odds ratio and its statistical significance

MI, myocardial infarction; PCI, percutaneous coronary intervention; TIMI, Thrombolysis in Myocardial Infarction; BARC indicates Bleeding Academic Research Consortium.
